# Curvefusion—A Method for Combining Estimated Trajectories with Applications to SLAM and Time-Calibration

**DOI:** 10.3390/s20236918

**Published:** 2020-12-03

**Authors:** Shitong Du, Helge A. Lauterbach, Xuyou Li, Girum G. Demisse, Dorit Borrmann, Andreas Nüchter

**Affiliations:** 1 College of Intelligent Systems Science and Engineering, Harbin Engineering University, Harbin 150001, China; lixuyou@hrbeu.edu.cn; 2 Informatics VII: Robotics and Telematics, Julius-Maximilians-University Würzburg, Am Hubland, 97074 Würzburg, Germany; helge.lauterbach@uni-wuerzburg.de (H.A.L.); dorit.borrmann@uni-wuerzburg.de (D.B.); 3 Faculty of Sciences, Technology and Communication, University of Luxembourg, 4, rue Alphonse Weicker, L-2721 Luxembourg; girumdemisse@gmail.com

**Keywords:** mapping, continuous-time SLAM, deformation-based method, time calibration

## Abstract

Mapping and localization of mobile robots in an unknown environment are essential for most high-level operations like autonomous navigation or exploration. This paper presents a novel approach for combining estimated trajectories, namely curvefusion. The robot used in the experiments is equipped with a horizontally mounted 2D profiler, a constantly spinning 3D laser scanner and a GPS module. The proposed algorithm first combines trajectories from different sensors to optimize poses of the planar three degrees of freedom (DoF) trajectory, which is then fed into continuous-time simultaneous localization and mapping (SLAM) to further improve the trajectory. While state-of-the-art multi-sensor fusion methods mainly focus on probabilistic methods, our approach instead adopts a deformation-based method to optimize poses. To this end, a similarity metric for curved shapes is introduced into the robotics community to fuse the estimated trajectories. Additionally, a shape-based point correspondence estimation method is applied to the multi-sensor time calibration. Experiments show that the proposed fusion method can achieve relatively better accuracy, even if the error of the trajectory before fusion is large, which demonstrates that our method can still maintain a certain degree of accuracy in an environment where typical pose estimation methods have poor performance. In addition, the proposed time-calibration method also achieves high accuracy in estimating point correspondences.

## 1. Introduction

Autonomous mobile robots have a wide range of applications, including planetary exploration, rescue, disaster scenarios and other tasks that are hazardous or unfeasible for humans. In unknown environments, localization and mapping are crucial tasks. Mobile mapping is defined as the data acquisition of the surrounding environment employing a mobile platform with several sensors. Data registration, i.e., merging the acquired scans or images into a common coordinate system, is an essential step in mobile mapping. The poses from the positioning sensors cannot be used directly due to imprecise measurements. Generally, four main sensors have been developed for robot localization, namely, global navigation satellite systems (GNSSs), inertial navigation systems (INSs), vision-based and laser-based methods. The GNSS is a simple and widely used solution for localization of outdoor robots [1]. However, some drawbacks, such as multipath errors, latency and lacking signal in indoor environments, limit its application [2]. INS acquires pose information by integrating inertial sensors, which are subject to unbounded accumulation errors due to bias and noise [3]. Vision-based methods can obtain robust and accurate motion estimation; however, they are vulnerable to ambient lighting conditions [4].

As an active sensor, the lidar is invariant to light. On the other hand, a typical 3D lidar, such as the Velodyne VLP-16, can acquire environmental information at around a 10 Hz scanning rate with a horizontal field of view (FOV) of 360 degrees and 30 (±15) degrees in the vertical direction. High resolution allows the lidar to capture a large amount of detailed information in an environment with long ranges. These advantages make lidar widely used in robot systems. Popular laser-based algorithms, such as Iterative Closest Point (ICP) [5] or Normal Distribution Transform (NDT) [6], use scan matching to estimate poses. Recently, semantic information has been integrated into scan matching, employing a deep neural network to improve registration [7].

Although the aforementioned methods can achieve excellent results, the pose estimation suffers from error accumulation in the long-term or large-scale scenes [8]. A solution is to fuse multiple sensors. Multi-sensor fusion techniques explore advantages of each sensor and compensate for drawbacks from one of them. The most common way is to integrate GNSS with INS [9]. Fusion strategies with vision or laser and inertial sensors have also become popular in recent years [10,11,12,13]. Bry [14] presents a novel extension of the Gaussian particle filter to estimate robot pose with an IMU (Inertial measurement unit) and a planar laser. In [15], a loosely coupled system based on an extended Kalman filter (EKF) combining stereo vision and IMU is proposed for pose estimation. However, updating all landmarks and the joint covariance matrix lead to a large computational burden with the number of landmarks.

Simultaneous localization and mapping (SLAM) utilizing the lidar or camera to generate a globally consistent mapping is a hot research area [16]. Typically, SLAM includes two parts, i.e., the frontend and backend. The frontend consists of an initial estimation of pose and data association. In the backend, a filtering or graph optimization framework is employed to further optimize the trajectory. In recent years, more and more researchers have tended to use graph optimization technology in the SLAM field. A graph-based network consists of nodes and edges. The nodes represent the pose information of the robot, while the edges reflect the mathematical relationship between adjacent nodes [17]. It is well known that many SLAM systems are equipped with multiple sensors. Moreover, autonomous driving data sets employ cameras, lidars and GPS/IMU for data acquisition, e.g., the KITTI [18] and the Malaga Urban [19] data sets. Time calibration and fusion methods are key steps in multi-sensor fusion SLAM.

This paper presents a curve shape-based method called *curvefusion* for combining estimated trajectories with applications to mobile mapping and time-calibration. There are four contributions in this paper. First, a coarse registration that combines two pose curves from 2D lidar and GPS to optimize poses of the planar three degrees of freedom (DoF) trajectory is carried out. While typical multi-sensor fusion methods mainly focus on the filter framework, we provide a new perspective, i.e., curve deformation, to deal with trajectory fusion. The proposed fusion method does not rely on a sensor model, i.e., we require only the trajectories themselves, which improves the computational efficiency.

Secondly, the output of curvefusion is fed into continuous-time SLAM, which is considered as a fine registration process. In [20], HectorSLAM is fed to continuous-time SLAM as an initial trajectory. Our system follows a similar framework. The difference is the initial trajectory of continuous-time SLAM, i.e., HectorSlam is replaced by curvefusion in our system. Since the essence of curvefusion is deformation, the trajectory after fusion is smoother than HectorSLAM. After applying continuous-time SLAM, a globally consistent 3D mapping is achieved.

Furthermore, a deformation-based time synchronization approach that does not rely on the timestamp and other hardware-based methods is proposed. We introduce the curve deformation in [21] into the time calibration problem of multiple sensors. The previous method is well suited to our problems without major changes, except in shape representation, which should be considered as a contribution. In addition, the proposed algorithms are in general applicable to trajectory optimization and time calibration and are not limited to SLAM and mobile robots.

**Figure 1:**
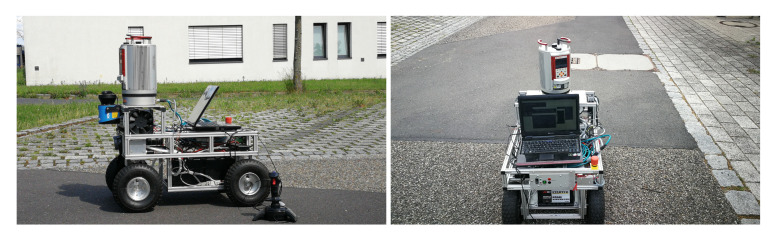
Images of the mobile robot mapping system.

The hardware platform for the proposed algorithm is shown in Figure 1, which system is equipped with a horizontally scanning SICK LMS100, a 3D laser scanner Riegl VZ-400 and a GPS module.

## 2. Related Work

There is an increasing body of scholarly work regarding autonomous vehicles with different mapping and time calibration solutions. In this section, we present a brief literature review that is related to our current work.

Traditional multi-sensor fusion methods are based on a filtering framework. Researchers have proposed a series of filtering algorithms in the inherent framework by using different sensors, improving dynamic models and state estimation methods [22]. However, the convergence of the updated probability distribution is not guaranteed. In addition, the difficulty of obtaining accurate sensor models and uncertainties are the main drawbacks of these methods [23]. In our approach, the fusion optimization problem of two trajectories is considered as the problem of deforming one curved shape to the other in a deformation space. To improve the accuracy of fused poses, a deformation-based multi-sensor fusion method is introduced in this paper. The proposed fusion method does not rely on the sensor model. As long as the trajectories of the sensor to be fused are given, we can easily obtain an optimized fusion trajectory, which greatly improves the computational efficiency compared to the filter-based sensor fusion method.

Time synchronization between different sensors is critical for multi-sensor fusion. Unfortunately, there is currently no explicit framework for time synchronization. Universal synchronization algorithms, such as the network time protocol (NTP), require the cooperation of each code, and are sometimes not applicable, for example, if one does not have access to the software embedded in a sensor, e.g., to the Riegl VZ-400 [24] used in this paper. In KITTI data sets [18], the GPS/IMU data were synchronized to the lidar time frame by their timestamp. Then, the nearest values were selected as the synchronization results. Olson [25] pioneered a passive synchronization method to reduce the synchronization error, like a clock correction-based approach. Recently, a pure software synchronization framework via ROS (Robot Operating System) nodes has been proposed to achieve low latency and low synchronization errors [26], but their system still works with the time queue using the nearest values. However, timestamps do not accurately represent the exact environmental information due to unknown sensor time delays. Moreover, if the sensor data are not from the same clock or a different processor, a timestamp-based method cannot be used to synchronize data. We present a novel deformation-based time synchronization approach that does not rely on the timestamp and other hardware-based methods.

SLAM technology has been widely applied to the robot community in recent years. Laser-based 2D SLAM techniques are a mature research area. Gmapping is currently the most widely used 2D slam method, which is a Rao-Blackwellized particle filter (PF) approach [27]. However, PF-based algorithms require a large number of particles to achieve excellent performance, which inevitably increases the computational complexity. In addition, this method relies on odometry and cannot be applied to drones and uneven areas. HectorSLAM does not require odometer information, which extends its application scenarios. HectorSLAM [28] represents the environment with a multi-resolution map, which avoids getting stuck into a local minimum. In a scan matching process, the newly acquired scan is matched with the existing map. The optimization of scan matching is solved using a Gauss–Newton approach. However, the method requires a lidar with a higher update frequency and low measurement noise. Hence, accurate mapping is often achieved when the robot speed is relatively low. Furthermore, the lack of loop close also leads to a large accumulation error. In [20], a backpack-mounted 3D mobile scanning system is proposed. In this system, HectorSLAM generates an initial trajectory of the backpack. The trajectory is then used to “unwind” the data of the Riegl VZ-400. Our system follows a similar framework. The difference mainly focuses on the acquisition of the initial trajectory. Instead of the single HectorSLAM, our algorithm utilizes a deformation-based method to fuse GPS and HectorSlam as the initial trajectory.

The frontend of SLAM involves data association and sensor pose initialization. Feature-based methods extract feature descriptors from two consecutive images or scans. These descriptors are then used to calculate point correspondences for relative pose estimates [29,30]. However, feature-based methods may fail in an unstructured environment, such as a highway. Another category is ICP, which works on the raw point-sets regardless of their intrinsic properties. ICP iteratively finds point correspondences between two consecutive point sets and minimizes a distance costfunction [5]. However, the algorithm relies on good initial pose guesses and may easily fall into a local minimum. Moreover, there is a drawback in the frontend estimation, i.e., incremental registration leads to the error accumulation. In the backend, a filtering or graph optimization framework is employed to further optimize the trajectory. In terms of the filtering method, extended Kalman filters (EKFs) [31] and particle filters (PFs) [27] are the most widely used technologies in the SLAM field. For an EKF, the observation update step needs to update all landmarks every time, which leads to an exponential increase in computational complexity with the number of landmarks. In addition, EKF is extremely vulnerable to incorrect data association. Unlike an EKF, a PF has no linear Gaussian assumption. By applying Rao-Blackwellization to reduce the sample space, this method makes it possible to directly use a particle filter to calculate high-dimensional state space problems in SLAM. However, the drawback is that a PF consumes more computing resources in large scenes with more particles [16].

In recent years, an increasing number of researchers have tended to use graph optimization technology in the SLAM field [32,33]. A graph-based network consists of nodes and edges. The nodes represent the pose information of the robot, while the edges reflect the mathematical relationship between adjacent nodes. The study of shapes has many applications in art, computer vision, engineering and bioinformatics. Arena et al. [34] demonstrate the universal role that cellular nonlinear networks (CNNs) play in shape analysis, which could be extended towards surfacefusion. Deformation has also been introduced into the slam field. Ref [35] proposed ElasticFusion, a seminal map-centric approach that builds a globally consistent map utilizing a deformation graph without a pose graph. Although ElasticFusion improves the global consistency of the map, some features in the algorithm, such as confidence-based fusion, cannot be extended to other sensor models beyond RGB-D. Inspired by ElasticFusion, a novel approach was proposed in [36], which fuses inertial measurements into the map. However, deformation is primarily used as a constraint to eliminate the drift from the inertial sensors. Furthermore, the authors of [37] presented an extension of the ElasticFusion SLAM algorithm to lidar sensors. However, non-rigid deformation is applied in the backend, i.e, global relaxation, not in the frontend.

Our algorithm still follows a general SLAM framework. In the frontend, a coarse registration that utilizes a deformation-based representation to obtain the fused pose of two sensors is proposed. The output of the frontend is fed into the backend, i.e, a continuous-time SLAM framework to achieve a globally consistent mapping.

## 3. Methodology

### 3.1. System Overview

**Figure 2:**
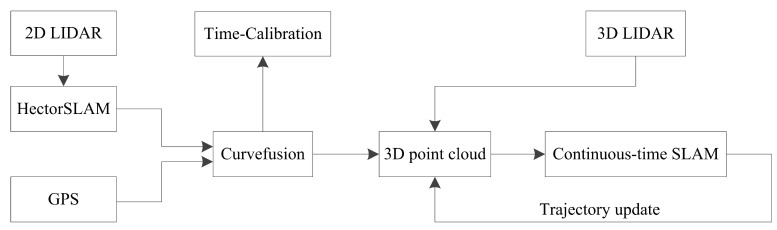
Overview of the proposed system architecture.

The architecture of the system is shown in Figure 2. First, the 2D lidar data are fed into HectorSLAM. The trajectories of HectorSLAM and GPS are then fused by curvefusion. After this step, the output and 3D lidar points serve as the input of the continuous-time SLAM framework. In addition, a time-calibration algorithm is then presented, which is another application of our curvefusion. The detailed algorithm principle will be introduced in the following sections.

### 3.2. Curvefusion

Shape representation based on deformation is the basis of our method. Curvefusion is actually the mathematical transformation of the shape representation. Therefore, an introduction about the shape representation is necessary.

#### 3.2.1. Shape Representation

In this section, a representation of curved shapes, adapted from [38], is introduced. The method represents a curved shape by finitely many rigid transformation matrices in the deformation space rather than a series of point coordinates. Previous publications have discussed shape representation in detail. However, the representation is mainly used for shape similarity metrics in computer graphics, which cannot be directly used to solve our problem. To clearly illustrate our method, we will re-discuss this shape representation and some improvements will be clearly mentioned here.

A curve S is represented by a series of coordinate points on the curve, i.e., $S=(p_{1},\cdots ,p_{k})$, where $p_{i}$ is a coordinate point, i.e., $p_{i}\in R^{n}$. C is defined as the set of all possible continuous curved shapes in $R^{n}$ approximated by *k* points, i.e., S∈C. In the previous work, the authors demonstrated that some deformations are not shape altering, such as translation and uniform scaling. These deformations preserve both the shape and the order of points, which cause redundancy in the shape similarity metric and need to be filtered out. In our problem, the curve is represented by a series of ordered position coordinates. Once the filtering procedure is done, the position and scale of each moment will be changed. Hence, the filter module in the original shape representation is removed.

After removing the filter procedure, the remaining part still follows the original framework. We define $\hat{G}=\left({\hat{g}}_{1},\cdots ,{\hat{g}}_{k}\right)\in SE{\left(n\right)}^{k}$. Here SE(n) denotes the special Euclidean group and SO(n) denotes the special orthogonal group. Consider the mapping function *f* defined on C as follows:

(1) $$\begin{array}{c} f\left(S\right)=\left\{\begin{array}{ccc} \hat{G} & =({\hat{g}}_{1},\cdots ,{\hat{g}}_{k}) & \mathrm{if}\;S\;\mathrm{is}\;a\;\mathrm{closed}\;\mathrm{curve}.\\ \hat{G} & =({\hat{g}}_{1},\cdots ,{\hat{g}}_{k-1}) & \mathrm{if}\;S\;\mathrm{is}\;\mathrm{an}\;\mathrm{open}\;\mathrm{curve}. \end{array}\right. \end{array}$$

such that

(2) $$\begin{array}{c} {\hat{g}}_{i}p_{i}=p_{i+1} \end{array}$$

where ${\hat{g}}_{i}$ is the transformation matrix between $p_{i}$ and $p_{i+1}$. Note the difference between open curves and closed curves. $S=(p_{1},\cdots ,p_{k})$ is defined as an open curve when $p_{1}\neq p_{k}$. A curve $S=(p_{1},\cdots ,p_{k})$ is a closed curve when $p_{1}=p_{k}$.

Assuming that a starting reference position $p_{1}$ and a fixed direction are available, $S=(p_{1},\cdots ,p_{k})$ are defined equivalently as

(3) $$\begin{array}{c} S=f^{-1}\left(\hat{G}\right)=\left(p_{1},{\hat{g}}_{1}p_{1},{\hat{g}}_{2}{\hat{g}}_{1}p_{1},\cdots ,\left(\prod_{i=1}^{k}{\hat{g}}_{i}\right)p_{1}\right) \end{array}$$

Equation (3) is for closed curves. We define open curves in a similar fashion:

(4) $$\begin{array}{c} S=f^{-1}\left(\hat{G}\right)=\left(p_{1},{\hat{g}}_{1}p_{1},{\hat{g}}_{2}{\hat{g}}_{1}p_{1},\cdots ,\left(\prod_{i=1}^{k-1}{\hat{g}}_{i}\right)p_{1}\right) \end{array}$$

Thus, $S=(p_{1},\cdots ,p_{k})$ can be represented by the corresponding $\hat{G}$. As a result, a final expression of the novel curved shape representation is defined in Equation (1).

**Figure 3:**
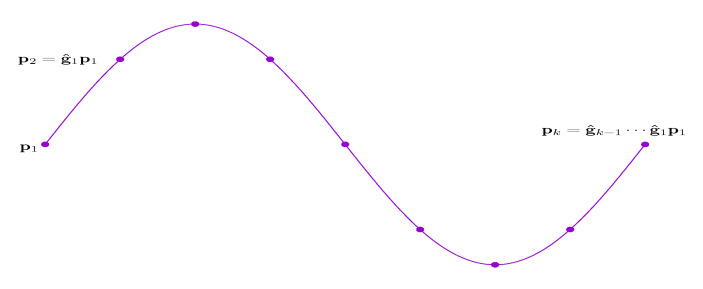
Given a fixed starting position $p_{1}$, the curve is reconstructed by the successive application of the transformations $({\hat{g}}_{1},\cdots ,{\hat{g}}_{k-1}).$

Consequently, a curved shape is represented by finitely many rigid transformation matrices, i.e., $\hat{G}$. Given a starting reference position and a fixed direction, the curved shape can be constructed using $\hat{G}$ according to Equations (3) or (4), cf. Figure 3.

To accurately represent the curved shape, it is necessary to obtain the optimal ${\hat{g}}_{i}\in SE\left(n\right)$ between two consecutive positions $p_{i},p_{i+1}\in R^{n}$. The optimal rotation matrix R is computed by first estimating the rotation plane and then using Equation (5). First, an orthonormal basis B is obtained from $p_{i}$ and $p_{i+1}$ using singular value decomposition (SVD); the plane spanned by the two eigenvectors with the largest eigenvalues is taken as the rotation plane. Next, R∈SO(2) is estimated by

(5) $$\begin{array}{c} \min_{R\in SO(2)}{∥R{\tilde{p}}_{i}-{\tilde{p}}_{i+1}∥}_{2}^{2}, \end{array}$$

where ${\tilde{p}}_{i}$ and ${\tilde{p}}_{i+1}$ correspond to the two-dimensional parts of $p_{i}$ and $p_{i+1}$, respectively. Further, R∈SO(2) is expressed in homogeneous coordinates as $R_{h}\in SO\left(n\right)$. Finally, the optimal rotation matrix R is given as

(6) $$\begin{array}{c} R=BR_{h}B^{T}. \end{array}$$

With the optimal rotation R, the optimal translation vector is determined by

(7) $$\begin{array}{c} t=p_{i+1}-Rp_{i}. \end{array}$$

#### 3.2.2. Trajectory Fusion

The approach developed in this paper extends the prior work of [21,38]. The previous work is designed for calculating the similarity metric of curved shapes. Specifically, a similarity metric with an explicit geodesic distance based on the shape representation in Section 3.2.1 is proposed. This aims to characterize the intermediate process of deforming one curved shape to the other. We regard the intermediate processes of deformation as fusion trajectories. Hence, the method is introduced to solve the trajectory optimization problem.

However, some improvements have to be made. First, the previous algorithm is only designed to characterize a deformation process. Therefore, the translation and uniform scaling are filtered out at the beginning, which we have mentioned in Section 3.2.1. In fact, the intermediate curved shape obtained in previous work is similar to Equation (1). By Equation (3) or (4), we can easily calculate the point representation of the curve. However, if the filter module is utilized, these reconstructed points do not represent the position information of the trajectory. To preserve location consistency, the filter module is not enabled in our algorithm.

Furthermore, after removing the filter module, the published algorithm can calculate a series of intermediate curves, but these curves only contain position information not rotation. We aim to obtain pose fusion instead of one of these. To address this, an approach to calculate the rotations of the fusion curves is presented, which is an extension of previous work.

Additionally, as mentioned above, the previous work aimed to characterize the intermediate process of deforming one curved shape to the other, which results in several intermediate curves. The goal of our algorithm is to obtain the optimal fusion curve. Hence, a selection criterion for the optimal intermediate curve is proposed in this paper. A detailed discussion is presented as follows.

Given two trajectories from the same system with different sensors or algorithms, e.g., GPS and HectorSLAM [28], our method regards these two trajectories as two curves that represent the same shape. The fusion optimization problem of two trajectories is then considered as the problem of deforming one curved shape to the other in a deformation space. In the process of deformation, several intermediate curves are generated. By selecting the optimal intermediate curve, the optimal fusion trajectory that combines the advantages of the two input curves is obtained. In the following we devise the algorithm using curves from GPS trajectories and HectorSLAM trajectories as an example. However, curvefusion is applicable in general setups. Next, we discuss how to combine two curve trajectories to get a new trajectory that includes poses. The following discussion mainly focuses on closed curves. The main idea of the algorithm is to calculate the geodesic path that shows how a curved shape deforms into another shape.

Assume there are two curves $S^{1}$ and $S^{2}$. As described in Section 3.2.1, every curve S∈C has a corresponding representation in $\hat{G}$. Thus, we can get ${\hat{G}}^{1}$ and ${\hat{G}}^{2}$:

(8) $$\begin{array}{c} {\hat{G}}^{1}=\left({\hat{g}}_{1}^{1},\cdots ,{\hat{g}}_{k}^{1}\right), \end{array}$$

(9) $$\begin{array}{c} {\hat{G}}^{2}=\left({\hat{g}}_{1}^{2},\cdots ,{\hat{g}}_{k}^{2}\right), \end{array}$$

where the homogeneous form of ${\hat{g}}_{i}$ is given as

(10) $$\begin{array}{c} {\hat{g}}_{i}=\left(\begin{array}{cc} R_{i} & \upsilon_{i}\\ 0 & 1 \end{array}\right),s.t.,R_{i}\in SO\left(n\right),\upsilon_{i}\in R^{n}. \end{array}$$

Geodesic curves in SE(n) between two points, are denoted as

(11) $$\begin{array}{cc} \beta {\left(t\right)}_{i} & =R_{i}^{1}{\left({\left(R_{i}^{1}\right)}^{-1}R_{i}^{2}\right)}^{t} \end{array}$$

(12) $$\begin{array}{cc} \alpha {\left(t\right)}_{i} & =\upsilon_{i}^{1}+\left(\upsilon_{i}^{2}-\upsilon_{i}^{1}\right)t, \end{array}$$

where t∈[0,1]. $R_{i}^{1}$ and $R_{i}^{2}$ represent the rotation parts of ${\hat{g}}_{i}^{1}$ and ${\hat{g}}_{i}^{2}$ respectively while $\upsilon_{i}^{1}$ and $\upsilon_{i}^{2}$ are the translation parts. Then, the geodesic curve connecting two points of two curves is defined as follows:

(13) $$\begin{array}{c} g{\left(t\right)}_{i}=\left(\begin{array}{cc} \beta {\left(t\right)}_{i} & \alpha {\left(t\right)}_{i}\\ 0 & 1 \end{array}\right), \end{array}$$

where $g{\left(t\right)}_{i}\in SE\left(n\right)$. By applying Equation (13), the geodesic curve, i.e., intermediate curve ζ(t) between $S^{1}$ and $S^{2}$ is given as follows:

(14) $$\begin{array}{c} \zeta \left(t,{\hat{G}}^{1},{\hat{G}}^{2}\right)=\left(g{\left(t\right)}_{1},\cdots ,g{\left(t\right)}_{k}\right), \end{array}$$

where *k* is the number of position coordinates in a curve. By setting *t*, the different intermediate curves are obtained. Note that *t* ranges from 0 to 1 and the denominator of *t* determines the number of intermediate curves. Equation (14) characterizes how $S^{1}$ deforms into $S^{2}$ with intermediate steps. ζ(t) represents an intermediate curve with finitely many rigid transformation matrices. Substituting ζ(t) into Equation (3), the intermediate curve represented by a series of position coordinates is reconstructed. Since several intermediate curves are obtained, a selection criterion for the optimal intermediate curve is necessary, which is the extension of previous work. Here, the position error of the start point and the endpoint of the fusion trajectory is used as the selection criterion of the optimal intermediate curve. Further discussions on the selection criterion will be presented in Section 6.1.

It should be noted that the intermediate curve reconstructed by Equation (3) only includes positional information but no orientation. $g{\left(t\right)}_{i}$ in ζ(t) is only a curved shape representation in space not including rotation. Subsequently, an approach to calculate the rotation of the curve is presented, which is an extension of previous work. Although the following discussion mainly focuses on the 2D space, it is easily extendable to 3D. Let $R_{i}^{2o}$ denote a 2D matrix that represents the orientation information for the *i*th point of $S^{2}$. $R_{i}^{2o}$ is considered a tangent vector of the corresponding ${\hat{g}}_{i}^{2}$. If ${\hat{g}}_{i}^{2}$, $R_{i}^{2o}$ and ${\hat{g}}_{i}^{1}$ are known, we can push the tangent vector $R_{i}^{2o}$ to the tangent space of $S^{1}$. Then, the orientation at ${\hat{g}}_{i}^{1}$ can be recovered. The pushforward to the tangent space of ${\hat{g}}_{i}^{1}$ is given by

(15) $$\begin{array}{c} P_{i}=R_{i}^{1}{\left(R_{i}^{2}\right)}^{T}\log\left({\left(R_{i}^{2}\right)}^{T}R^{2o}\right). \end{array}$$

Consequently, the orientation at ${\hat{g}}_{i}^{1}$ is calculated by

(16) $$\begin{array}{c} R_{i}^{1o}=R_{i}^{1}\exp\left(P_{i}\right). \end{array}$$

Subsequently, if the starting point $p_{1}$ is known, the positions of the intermediate curves are computed by using Equation (3), while the orientation is obtained by Equations (15) and (16). Thus, by selecting the optimal intermediate curve, the optimal fusion trajectory that combines the advantages of the two input curves is obtained.

## 4. Continuous-Time SLAM

The fusion trajectory from our curvefusion algorithm is fed into the continuous-time SLAM to further improve the trajectory and the 3D map quality. Specifically, the output of curvefusion is used to discretize time and provide an initial trajectory for the continuous-time SLAM. More mathematical details of our continuous-time SLAM algorithm are are given in [39]. To clearly discuss the application of curvefusion in this part, the basic idea of the continuous-time SLAM is summarized as follows.

This method is similar to ICP. The corresponding point is found using the nearest neighbor search, and the optimal trajectory is estimated by iteration. The difference of continuous-time SLAM is that it was developed for mobile mapping where no separate terrestrial 3D scans exist. In mobile mapping, applying rigid pose estimation to this non-rigid problem directly is problematic. A solution is to coarsely discretize the time. This results in a partition of the trajectory into sub-scans that are treated rigidly. Then, rigid registration algorithms, like the ICP and other solutions to the SLAM problem, are employed. Obviously, trajectory errors within a sub-scan cannot be improved in this fashion.

For the continuous-time SLAM, a much finer discretization of the time, at the level of individual 2D scan slices or individual points, is utilized. In this paper, time is discretized according to the update frequency of curvefusion, which is consistent with HectorSLAM. The robot moves from $t_{0}$ to $t_{n}$, generating a trajectory $T=(V_{0},\cdots ,V_{n})$, where $V_{i}$ denotes the pose of the robot at moment $t_{i}$. $M=(m_{0},\cdots ,m_{n})$ is the set of lidar measurements, where $m_{i}=\left(m_{x,i},m_{y,i},m_{z,i}\right)$ is a point acquired at $t_{i}$ in the local coordinate system of $V_{i}$. If $V_{i}$ represents more than one point, $V_{i}$ is assigned to the first point and the remaining points are motion compensated using pose interpolation. $P=(p_{0},\cdots ,p_{n})$ represents the points set in the global coordinate frame. Given M and T, $p_{i}$ is calculated by $p_{i}=V_{i}⊕m_{i}=R_{i}*m_{i}+t_{i}$. The optimal trajectory $\hat{T}=\left({\hat{V}}_{0},\cdots ,{\hat{V}}_{n}\right)$ is obtained so that P generated via $\hat{T}$ more closely resembles the real environment.

For each $p_{i}$, the closest point $p_{j}$ is found via the nearest neighbor search based on $|t_{i}-t_{j}|>\delta$, where δ is again the minimal amount of time that must have elapsed for the laser scanner to have measured the same point again, that is, the point matching procedure starts to run only when the time interval between two points is greater than δ. After the corresponding points are established, the pose differences and their respective covariances are calculated, $\hat{T}$ is optimized iteratively. This process is stopped until the change in the trajectory falls below a threshold. The positional error of two poses, $V_{i}$ and $V_{j}$, is defined as follows:

(17) $$\begin{array}{c} E_{i,j}=\sum_{k=i-N}^{i+N}{∥V_{i}⊕m_{k}-V_{j}⊕m_{k}^{{}^{\prime }}∥}^{2} \end{array}$$

where $m_{k}$ and $m_{k}^{{}^{\prime }}$ are the corresponding point pairs, and *N* represents a small neighborhood of points taken in the order of hundreds of milliseconds that is assumed to have negligible pose error.

It can be seen from the above that continuous-time SLAM needs an initial trajectory *T* and a time-discrete method. In [20], the initial trajectory is determined by HectorSLAM. Here, we use cuvefusion to integrate GPS with HectorSLAM as the initial trajectory *T*. Time is discretized according to the update frequency of curvefusion.

## 5. Time Calibration

Data synchronization between different sensors is critical for multi-sensor fusion. Two strategies exist for time synchronization. First, a hardware trigger is used for data acquisition at the expense of requiring additional cables. However, even in the presence of a hardware trigger, one does not know when the sensors have acquired the data due to unknown delays within the sensors, for example a laser scanner might need to wait for a mirror rotation. The second approach is to set the sensors into a continuous data streaming mode and record the data using time stamping. Time stamping requires all recording devices to be synchronized, which is sometimes not possible, and even if it is possible, the timestamps do not accurately represent real-time environmental information due to sensor time delays.

In this section, a point correspondence estimation method in computer vision for the parametrization of curves is applied to our data synchronization. The method is proposed in [21] for point correspondence between two curved shapes, which is a prerequisite of the calculation of shape similarity. Since this method can easily be adopted to solve the time calibration problem, we just keep the complete original model. However, as far as we know, there is no relevant literature that considers the time calibration problem from the view of the curve shape. Next, we will present in detail how the method is applied to time calibration.

Now, assume there are two curves, $S^{1}$ which consist of *k* positions in a fixed order and $S^{2}$ with *l* positions respectively, where k<l. Since the order of the positions on the curve is fixed, the time calibration between two curves can be considered as finding the optimal samplers that are described as follows:

(18) $$\begin{array}{cc} S_{a}^{1} & =S^{1}\circ \xi_{a}, \end{array}$$

(19) $$\begin{array}{cc} S_{b}^{2} & =S^{2}\circ \xi_{b}, \end{array}$$

where $\xi_{a}$ and $\xi_{b}$ represent the optimal samplers for $S^{1}$ and $S^{2}$, respectively, $S_{a}^{1}$ and $S_{b}^{2}$ are the curves that are optimally sampled with corresponding points.

Since the reading frequency of the GPS receiver is lower than the laser scanning rate, i.e., k<l, the point correspondence estimation based on optimal sampling for the calculation of shape similarity is applied to our problem. The main idea is to fix curve $S^{1}$ as a reference that is represented by $f\left(S^{1}\right)=\left({\hat{g}}_{1}^{1},\cdots ,{\hat{g}}_{k}^{1}\right)$, and then find the optimal sampler $\xi_{b}$ of $S^{2}$, with respect to $S^{1}$. Please note that a series of the mathematical operations in this part is based on Section 3.2.1 and Section 3.2.2. The general form is described as

(20) $$\begin{array}{c} \left.S^{2}\circ \xi_{b}=\left(p_{1}^{b},p_{2}^{b},\cdots ,p_{k}^{b}\right)\right.\;p_{i}^{b}\in U_{i} \end{array}$$

where $p_{i=1,\cdots ,k}^{b}$ are the sampled positions using sampler $\xi_{b}$, and the size of $U_{i=1,\cdots ,k}$ denotes the search space of $p_{i}^{b}$. The whole curve $S^{2}$ is covered by sliding a window along the trajectory to get $U_{i=1,\cdots ,k}$. The optimal sampler $\xi_{b}$ is obtained by optimizing Equation (21), which is written as follows:

(21) $$\begin{array}{c} \arg\min_{\xi_{b}}\sum_{i=2}^{k}\phi_{i}\left(p_{i-1}^{b},p_{i}^{b}\right), \end{array}$$

where $\phi_{i}$ is defined as

(22) $$\begin{array}{c} \phi_{i}\left(p_{i-1}^{b},p_{i}^{b}\right)=\alpha d{\left({\hat{g}}_{i}^{1},{\hat{g}}_{i}^{2}\right)}^{2}+\beta \left(A_{S^{2}}\left(p_{i-1}^{*},p_{i}^{*}\right)-A_{S^{2}}\left(p_{i-1}^{b},p_{i}^{b}\right)\right), \end{array}$$

where $p_{i-1}^{b}$ and $p_{i}^{b}$ are sampled positions of $S^{2}$ using a candidate sampler for the (i−1)th and *i*th positions, such that ${\hat{g}}_{i}^{2}p_{i-1}^{b}=p_{i}^{b}$, $p_{i-1}^{*}$ and $p_{i}^{*}$ are sampled points of $S^{2}$ for the (i−1)th and *i*th positions by a uniform sampler $\xi_{*}$. Generally, $d{\left({\hat{g}}_{i}^{1},{\hat{g}}_{i}^{2}\right)}^{2}$ is the cost functional that is used to calculate the point matching between two curves. However, the cost functional assumes the sampling functions preserve shape. On the contrary, if the sampling function does not preserve shape then the result will deviate from the target shape. To this end, a constrained objective functional A(·) that attempts to enforce shape preservation is added to $d{\left({\hat{g}}_{i}^{1},{\hat{g}}_{i}^{2}\right)}^{2}$. A(·) is a function that computes the area of a given curve in $R^{2}$, which is a strong shape preservation constraint. Assume $S=(p_{1},\cdots ,p_{k})$, $A_{S}$ is defined as follows:

(23) $$\begin{array}{c} A_{S}=\frac{1}{2}\sum_{j=1}^{k}\left(p_{j}^{y}-p_{(j+1)modk}^{y}\right)\left(p_{j}^{x}-p_{(j+1)modk}^{x}\right) \end{array}$$

where $p_{j}^{x}$ and $p_{j}^{y}$ denote the *x* and *y* coordinate components of the positions $p_{j}$. Note that $A_{S^{2}}\left(p_{i-1}^{*},p_{i}^{*}\right)$ is evaluating Equation (21) per sequential points. Thus, Equation (22) is obtained. α and β are weighting factors. ${\hat{g}}_{i}^{1}$ and ${\hat{g}}_{i}^{2}$ are representatives of the corresponding points of the curves $S^{1}$ and $S^{2}$, respectively. $d({\hat{g}}_{i}^{1},{\hat{g}}_{i}^{2})$ is the geodesic distance connecting two points of two curves that is given in the following form:

(24) $$\begin{array}{c} d\left({\hat{g}}_{i}^{1},{\hat{g}}_{i}^{2}\right)=(∥\log\left({\left(R_{i}^{1}\right)}^{T}R_{i}^{2}\right){∥}_{F}^{2}+∥\upsilon_{i}^{2}-\upsilon_{i}^{1}{{∥}_{2}^{2})}^{1/2} \end{array}$$

where $R_{i}^{1}$ and $R_{i}^{2}$ represent the rotation parts of ${\hat{g}}_{i}^{1}$ and ${\hat{g}}_{i}^{2}$, respectively, while $\upsilon_{i}^{1}$ and $\upsilon_{i}^{2}$ are the translation parts. Please refer to Equation (12).

Here, a procedure relating to how curvefusion is applied to time-calibration is described. Since the mathematical operations involved in this part are based on Section 3.2.1 and Section 3.2.2, we regard this part as a direct application of curvefusion. Assume there are two curves: $S^{1}$, which consists of *k* position coordinates in a fixed order, and $S^{2}$ with *l* position coordinates, where k<l. The main idea is to fix curve $S^{1}$ as a reference and then find the optimal sampler $\xi_{b}$ of $S^{2}$ with respect to $S^{1}$. Hence, the problem of time calibration is transformed into obtaining the optimal sampler $\xi_{b}$ by minimizing Equation (21).

## 6. Experimental Results

### 6.1. Trajectory Fusion Evaluation

**Figure 4:**
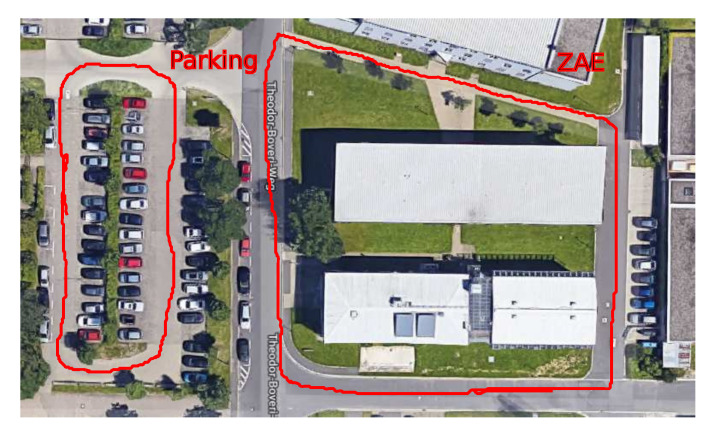
The corresponding satellite images for the Bavarian Center for Applied Energy Research (ZAE) and parking lot data sets. Red curves are the trajectories the robot traveled [40].

To evaluate the performance of the proposed approach, two data sets from the campus of Julius Maximilian University of Würzburg (Bavarian Center for Applied Energy Research (ZAE) and parking lot) were collected by our mobile robot, Achim3D. Achim3D is a VolksBot robot with Ackermann-like steering and is equipped with a horizontally scanning SICK LMS100, the high-end 3D laser scanner Riegl VZ-400 and a GPS module. In the first data set, the robot was driven starting in front of the robotics hall around the old ZAE building (Bavarian Center for Applied Energy Research). The complete loop took 429 s and was 280 m long. The trajectory around the parking lot was acquired as the second data set, which was 150 m long and took 258 s. Figure 4 is the satellite map of the two data sets.

To improve the accuracy of the 3D point cloud, we combined the trajectories from HectorSLAM and GPS by applying our curvefusion approach. Note that the outputs of both HectorSLAM and curvefusion are 2D trajectories. In the first part of the experiments, the results of HectorSLAM and curvefusion were used to “unwind” the data of the Riegl VZ-400. Some intuitive 3D point cloud displays are presented, which will show that our curvefusion outperforms HectorSLAM.

In the second part of experiments, HectorSLAM and curvefusion were first fed into the continuous-time SLAM. Then the output trajectories were used to “unwind” the data of the Riegl VZ-400. Similarly, some intuitive 3D point cloud displays are presented, which will show that our curvefusion + continuous-time SLAM outperforms HectorSLAM + continuous-time SLAM. In addition, some quantitative error analysis relative to the ground truth using cloudcomare is also presented in this part. In this paper, the ground truth is obtained with *3DTK—The 3D Toolkit* [41], which is designed for the automatic high-precise registration of terrestrial 3D scans, i.e., globally-consistent scan matching. When the robot equipped with the Riegl VZ-400 moves along the same environments, we enable a scan-go-stop fashion to collect several terrestrial laser scans, which are registered with *3DTK—The 3D Toolkit*. Here, 12 terrestrial 3D laser scans were acquired around the old ZAE building and the parking lot.

**Figure 5:**
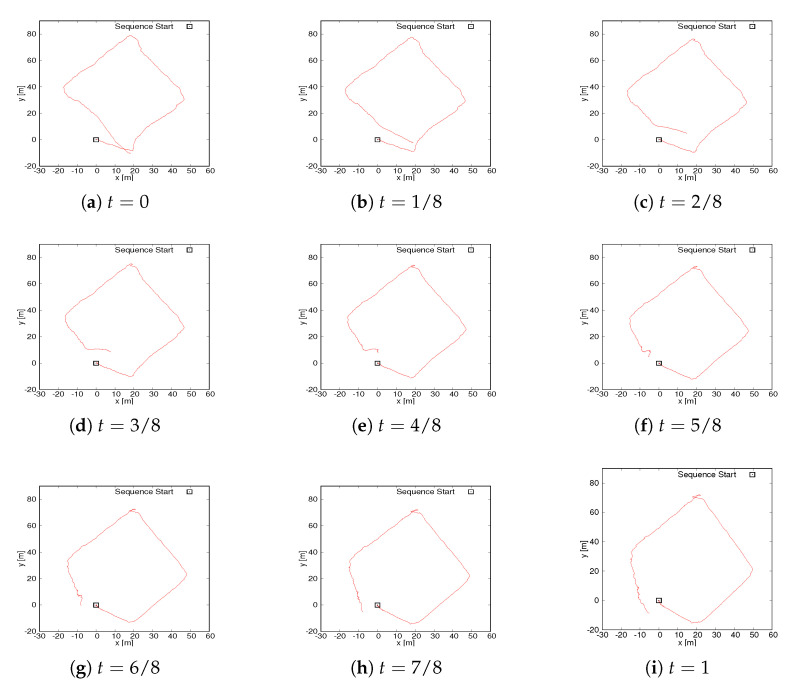
Fused trajectories. The box marks the starting point. t=0: original GPS trajectory. t=1: initial trajectory from HectorSLAM. The rest of the figures are a series of fusion trajectories. All curves are from the ZAE data sets.

Before carrying out the experiments, some questions about the intermediate curve are first discussed. As discussed earlier, several intermediate fusion curves were obtained in Section 3.2.2. Figure 5 shows the intermediate curve results from the ZAE data set. By setting *t* in Equation (14), the different intermediate curves are obtained. Note that *t* ranges from 0 to 1 and the denominator of *t* determines the number of intermediate curves. The larger the denominator of *t*, the more intermediate curves, which will increase the calculation burden of the system, and few intermediate curves cannot fully highlight the characteristics of fusion curves. In our experiments, we set the intermediate curve as eight, which means that *t* changes from 0/8 to 8/8. Here, the position error between the start point and the endpoint of the fusion trajectory is used as the selection criterion of the optimal intermediate curve. Specifically, the position error of a certain intermediate curve is minimal, we assume that the curve is the optimal fusion curve, which also intuitively measures the degree of closure of a loop. As seen from Figure 5, for the pure HectorSLAM trajectory, i.e., t=1, the endpoint lies outside of the trajectory polygon due to error accumulation, while curves (b)–(d) have the endpoint inside the polygon and are more similar to the GPS trajectory. According to the selection criterion, t=4/8 is the optimal fusion curve in the ZAE data sets and t=3/8 is the optimal fusion curve in the parking lot data sets. To demonstrate the performance of the proposed approach, curves (d)–(h) are also selected as fusion curves.

**Figure 6:**
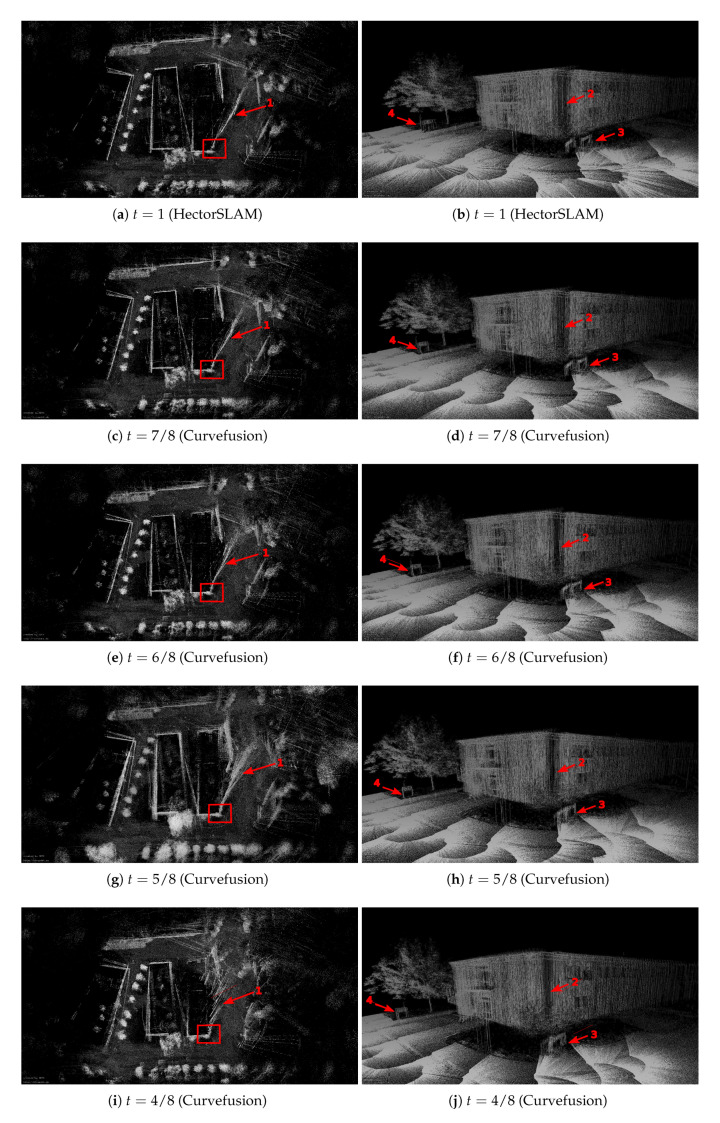

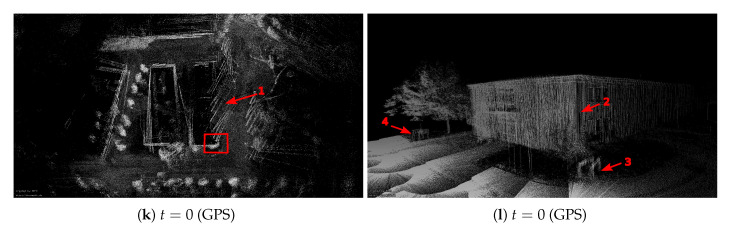
Visual inspection of the ZAE data sets. Left column: 3D point cloud results from bird’s eye view. Right column: the corresponding perspective views. The right column shows perspective views that correspond to the areas marked with red rectangles in the left column.

Figure 6 compares the 3D point cloud result using only the HectorSLAM trajectory with the trajectories from our curvefusion approach from the bird’s eye and perspective views of the ZAE data sets. The 3D point cloud results are generated by using the trajectories to unwind the data of the Riegl VZ-400, where t=0 refers to GPS trajectory and t=1 represents HectorSLAM trajectory. The larger *t* indicates the fusion curve is closer to HectorSLAM. The left column visualizes results from the bird’s eye view while the right column shows perspective views that correspond to the loop closure in the left column, i.e., the areas marked with red rectangles in the left column. The red arrows mark some examples for the improvements by our curvefusion approach.

For the ZAE data set, the quality of the point cloud from the pure HectorSLAM trajectory suffers from large errors, especially at the loop closure, i.e., Arrow 1 in Figure 6a. As the marked areas with Arrow 2 in Figure 6b show, a large gap occurs at the intersection of the two walls of the building and the street signs (Arrows 3 and 4) in the real scene are mistakenly turned into two. When t=7/8, i.e., Figure 6c,d, the large gap marked by Arrows 1 and 2 is slightly closed and the street signs are slightly revised compared to HectorSLAM. However, the performance does not improve significantly since the curve is so close to the original HectorSLAM trajectory, thus preserving many of the characteristics of the HectorSLAM trajectory including the errors. As *t* decreases, which indicates the curve is closer to the GPS trajectory, the quality of the point cloud improves. When t=4/8, the large gap (Arrow 1) in Figure 6i is closed correctly and the rotation errors at the street signs (Arrows 3 and 4) in Figure 6j are eliminated. When t=0, the large gap (Arrow 1) in Figure 6k is closed, however, the border is also largely missing. Moreover, the street signs (Arrows 3 and 4) in Figure 6l remain rotated. Consequently, our fusion curve t=4/8 achieves the optimal result in the ZAE data set.

**Figure 7:**
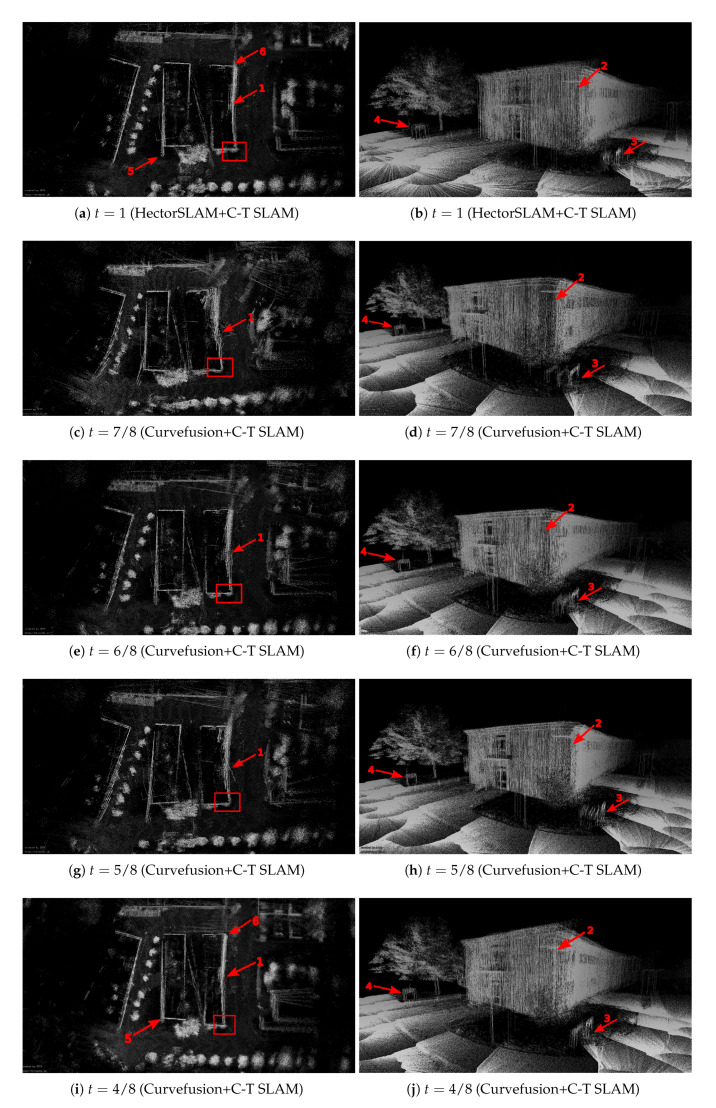

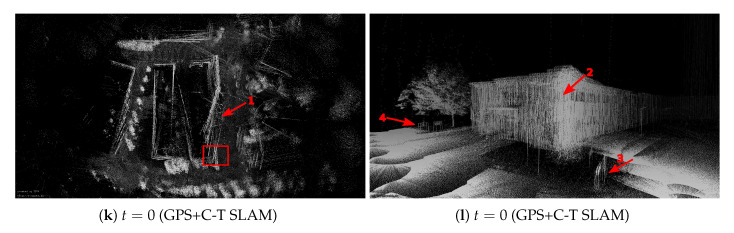
HectorSLAM + C-T SLAM and curvefusion + C-T SLAM denote HectorSLAM and curvefusion are first fed into the continuous-time SLAM. Then the output trajectories are used to unwind the data of the Riegl VZ-400. GPS + C-T SLAM has a similar definition.

After applying continuous-time SLAM, a descent map quality is achieved. Figure 7 shows visual results after running continuous-time SLAM for a fixed number of iterations. Here, HectorSLAM and curvefusion are first fed into the continuous-time SLAM. Then the output trajectories are used to unwind the data of the Riegl VZ-400. Compared with Figure 6, the gaps, i.e., Arrow 1 in the left column of Figure 7, are completely closed regardless of our fusion approach or the HectorSLAM trajectory. The corresponding 3D views of the red rectangle in the left column are shown in the right column, where two walls of this building are well connected (Arrow 2). As Figure 7b shows, the optimized point cloud quality of the HectorSLAM trajectory still suffers from large errors, e.g., the street signs (Arrows 3 and 4). Instead, our fusion approach eliminates the rotational error cf. Figure 7d,f,h,j, though the street sign (Arrow 3) remains slightly rotated. By comparison, t=4/8, i.e., Figure 7i,j, achieves the optimal results among our fusion trajectories. Compared with Figure 7a, some errors in corners (Arrows 5 and 6) are corrected completely. In conclusion, although point cloud quality both from our fusion trajectories and HectorSLAM have been significantly improved after applying continuous-time SLAM, our fusion approach achieves higher point cloud accuracy both locally and globally.

**Figure 8:**
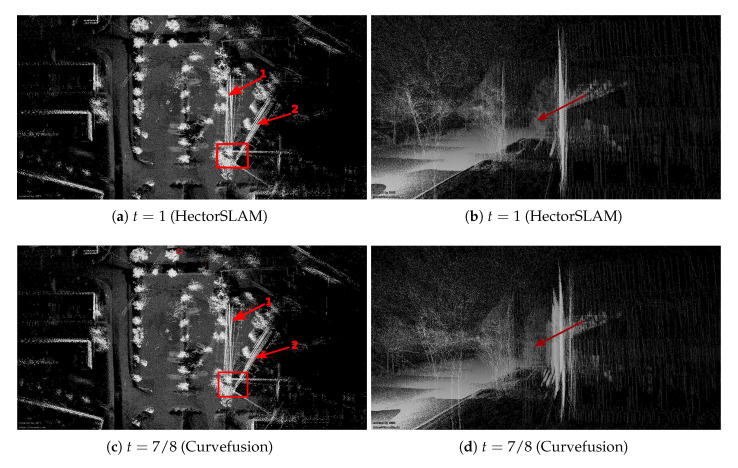

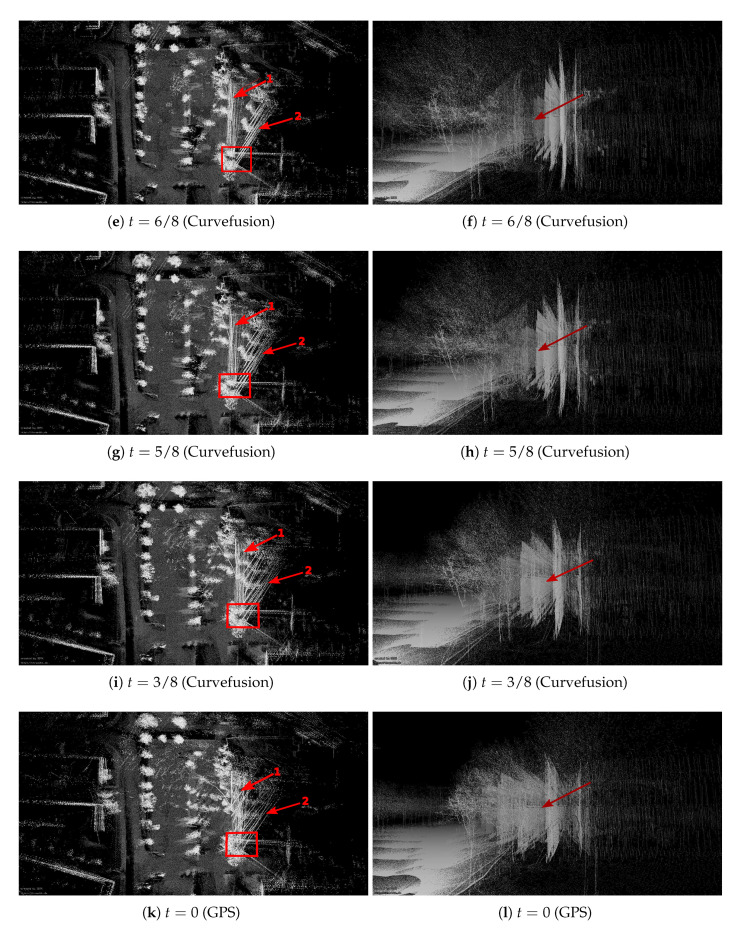
Visual inspection of the parking lot data sets. Left column: 3D point cloud results from bird’s eye view. Right column: corresponding perspective views marked with the red rectangle on the left column.

**Figure 9:**
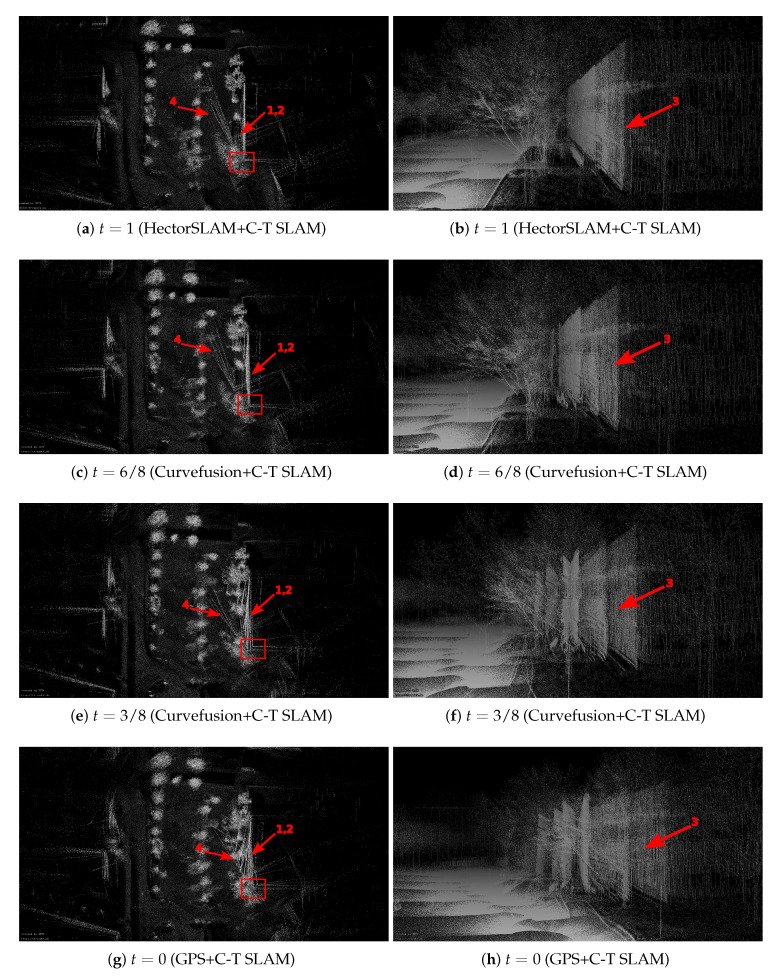
The results are optimized by continuous-time SLAM, and only four trajectories are selected.

Figure 8 and Figure 9 show results similar to Figure 6 and Figure 7, but from the parking lot data set. Figure 8 shows visual inspections using only the HectorSLAM trajectory and the trajectories from our curvefusion approach, where the Arrows 1 and 2 in the left column indicate the starting position and the final position, respectively. The right column corresponds to the red rectangle in the left column, i.e., the loop closure. As Figure 8a shows, the start and end points are far apart due to errors. Figure 8b is the corresponding 3D view, which shows a large gap (red arrow). With t=5/8, the rotation between Arrows 1 and 2 is slightly improved cf. Figure 8g and the gap (Figure 8h) is slightly smaller with respect to Figure 8a,b). When t=3/8, i.e., Figure 8i,j, the gap between Arrows 1 and 2 is further closed compared to Figure 8a,b. Compared with t=0, i.e., the pure GPS, Figure 8i,j, in t=3/8 achieves better results, although the results are only slightly better with respect to pure GPS.

Overall, our fusion method improves the quality of the point cloud, especially in the final part of the curve, where the loop closure happens. The gap caused by the trajectory error from HectorSLAM is closed or improved after using our fusion trajectory, since GPS does not suffer from global error accumulation.

**Figure 10:**
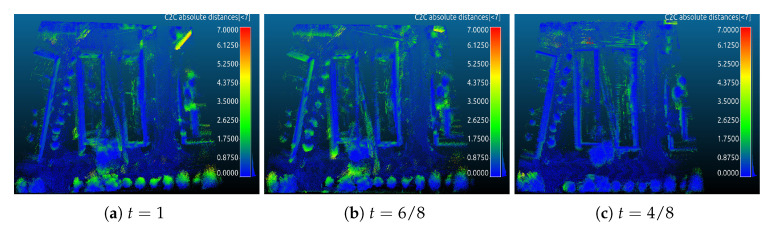
The visual inspection of computed errors from the ZAE data set: (**a**) indicates the result of pure HectorSLAM + continuous-time SLAM and (**b**,**c**) are from our curvefusion + continuous-time SLAM. Compared to Figure 7, only the three best results are given.

Figure 9 shows visual results after running continuous-time SLAM for the parking lot data set. Compared with Figure 8, the gaps between Arrows 1 and 2 are closed completely regardless of our fusion approach or HectorSLAM trajectory, cf. the left column. However, an additional rotation error has occurred (Arrow 4) with respect to Figure 8. Compared with Figure 9a, the rotation errors (Arrow 4) are improved cf. Figure 9e. From the 3D view, Figure 9b (Arrow 3), i.e., pure HectorSLAM, outperforms our fusion result, cf. Figure 9d,f. However, the comparison results with the ground truth in Figure 10 and Table 1 show that our fusion trajectories achieve higher accuracy. For reference, which we define ground truth as several terrestrial laser scans acquired with the Riegl VZ-400 and registered with *3DTK—The 3D Toolkit* [41].

**Figure 11:**
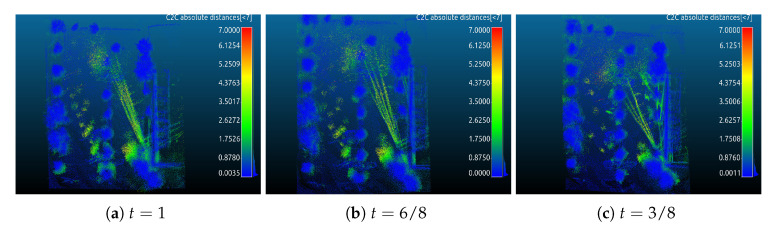
Same as Figure 10 but for parking lot data set.

Cloud to cloud distance error is used to evaluate the accuracy of point clouds by CloudCompare [42]. Figure 10 and Figure 11 are visual inspections of the ZAE and parking lot data sets, respectively. Yellow indicates high point to point distances and blue colors represent low errors. The color scale ranges from 0 to 12 m. The errors of point clouds optimized by continuous-time SLAM to ground truth are given in Table 1. As the results show, for the ZAE data set, t=4/8 shows better results than others, cf. Figure 7 and Figure 10c and Table 1. t=3/8 achieves the optimal point cloud quality in the parking lot data set, cf. Figure 9 and Figure 11c and Table 1.

### 6.2. Time-Calibration Evaluation

Time synchronization is performed to obtain the correspondence of data representing the same attributes from different sensors. These attributes mainly refer to direct or indirect position or attitude information. Sensor data acquisition suffers from time delays during the data transfer. In scenarios where more than one clock is available, synchronization of time stamps helps to improve the results. A typical example is our hardware platform, i.e., Achim3D, where all sensors such as the GPS, 2D laser scanner, IMU and the 3D laser scanner Riegl VZ-400 are connected to the same roscore, thus receiving ROS time stamps. However, the Riegl has its own clock, that is, the Riegl clock and the ROS clock are unrelated. Several mathematical models are used to synchronize the data of Riegl and other sensors [39].

**Figure 12:**
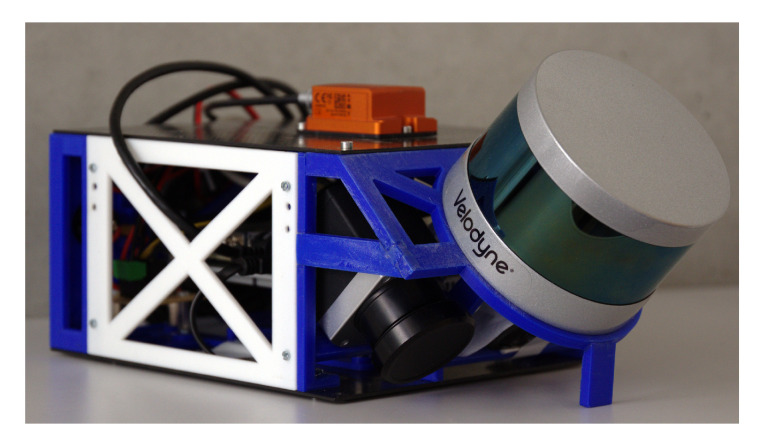
Prototype of the mapping module.

**Figure 13:**
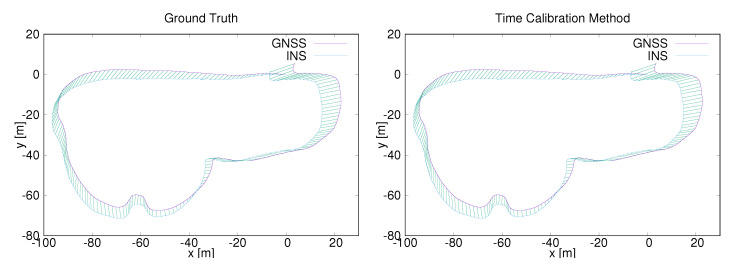
Data synchronization results. (**Left**): Synchronizing data by GPS timestamps (ground truth). (**Right**): Synchronizing data by the proposed time-calibration method. The trajectory is from the computer science building data set.

In this part, four data sets are used to test our time calibration method using curvefusion. One of them was collected from a UAV (Unmanned Aerial Vehicle) sensor payload featuring a GNSS and a lightweight laser scanner, cf. Figure 12. The payload consists of a Velodyne VLP16 Lite laser scanner. For positioning and localization, we mounted an XSens Mti-G 700. The trajectory was acquired around the computer science building [43]. The GPS trajectory and integrated position information from the IMU are synchronized by timestamp, which is also considered as ground truth in Figure 13. The left column shows the ground truth of point correspondence, while the right is calculated by our time-calibration method.

**Figure 14:**
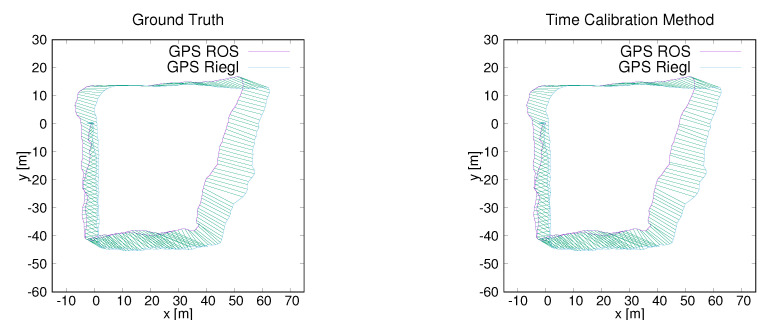
Data synchronization results. (**Left**): Synchronizing data by ground truth. (**Right**): Synchronizing data by the proposed time-calibration method. The trajectory is from the ZAEG data set.

**Figure 15:**
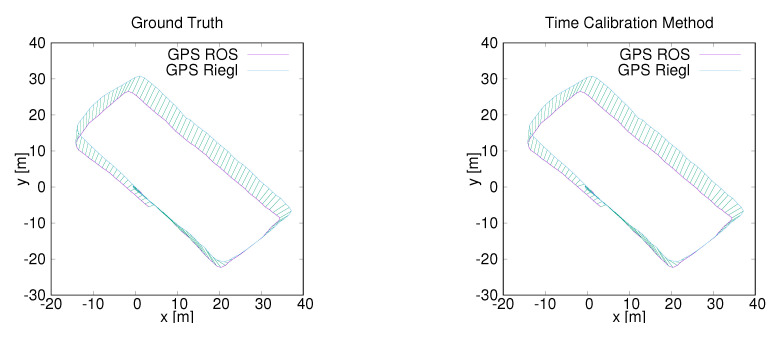
Data synchronization results. (**Left**): Synchronizing data by ground truth. (**Right**): Synchronizing data by the proposed time-calibration method. The trajectory is from the ParkingG data set.

**Figure 16:**
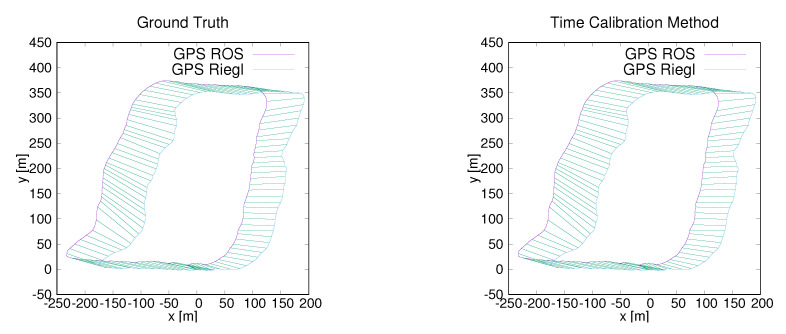
Data synchronization results. (**Left**): Synchronizing data by timestamps (ground truth). (**Right**): Synchronizing data by the proposed time-calibration method. The trajectory is from the Campus data set.

To further test the performance of the proposed method on our platform, we carried out two simplified experiments where two GPS modules were mounted, one on the robot and one connected to the Riegl, both having their own clock. Two data sets around the old ZAE building and the parking lot near the computer science building were collected by our hardware platform. The synchronized data from the two experiments is displayed in Figure 14 and Figure 15. To verify the performance of our time calibration method over long distances, the fourth data set was acquired around the campus. The first author walked around the campus in a large circle that took around 16 min and the total length was 1.3 km. In this experiment, two GPS modules were connected to two different computers which were put into a backpack. The visual display of the synchronized data is shown in Figure 16.

Intuitively, compared with the ground truth, our time calibration method achieves high point correspondence accuracy. To be able to quantitatively demonstrate the accuracy of the proposed method, some numerical values, i.e., mean, standard deviation and root mean square error (RMSE) of our time calibration results to the ground truth are given in Table 2. Table 2 shows that with correctly set parameters α and β in Equation (22), our time calibration method achieves a high accuracy on synchronizing data from different sensors. Parameter α is set to 1 according to [21]. For parameter β, assigning a large value leads to an objective functional that favors samplers that preserve area even with a high deformation cost and vice versa for a small β. However, large β also means point matching is not very elastic. If β is small, a highly flexible matching result is obtained but it might lose the original geometric moment of the curve $S^{2}$. In our experiment, β ranges from 0 to 1.

## 7. Conclusions and Future Work

We have presented a novel approach called curvefusion for combining estimated trajectories with applications to SLAM and time-calibration. The state-of-the-art for SLAM methods mainly focuses on pose graphs or probabilistic methods, whereas our approach instead adopts a deformation-based method to optimize the map. The fusion trajectory output from our curvefusion algorithm is then fed into continuous-time SLAM to further improve the trajectory and the 3D map quality. Furthermore, a novel deformation-based time synchronization approach that does not require timestamps was presented.

Experiments carried out with a mobile robot, equipped with a horizontally scanning laser scanner, a GPS module and a high-end 3D laser scanner as well as with a UAV sensor payload featuring a GNSS and a lightweight laser scanner, showed that the proposed approach achieves high 3D map quality and accurate time synchronization results.

Some factors that affect accuracy improvement have to be explained. Typically, GPS is more accurate on the global scale, while HectorSLAM is more accurate on the local scale. Hence, the loops close better by curvefusion. However, 2D lidar LMS100 is mainly applied in indoor environments, hence, HectorSLAM inevitably outputs trajectories with large errors. Furthermore, the lack of a loop eventually leads to a large gap between the starting position and end point, which is especially bad in outdoor environments. For the above reason, the final fusion result has not been significantly improved. However, one advantage of curvefusion is that even if the two worst trajectories are fused, they will be improved to a certain extent, which shows that our method can still maintain a certain accuracy in harsh environments.

In terms of fusion, GPS only contains position information, so that the final fusion result improves the accuracy in terms of position, but the fusion of rotation is actually a mapping of HectorSLAM pose information. In this case, the large pose error of HectorSLAM will have a negative impact on the fusion trajectory. In future work, we will consider the full integration of the position and attitude information of the two curves, e.g., combing visual odometry and lidar.

For the optimal curve selection criteria, when the position error between the start point and the endpoint of a certain intermediate curve is minimal, we think that the curve is the optimal fusion curve. This criterion is only applicable to scenarios with loops. The optimal fusion curve selection without loops is the problem we have to solve in the future.

In addition, since the proposed time calibration method is based on curve deformation, the trajectory shape of each sensor needs to be similar before calibration. If a curve has a large error, the final time calibration result is not satisfactory, however, this does not affect the practical significance of the proposed method since this precondition is not difficult to implement in the current popular trajectory algorithm.

## Figures and Tables

**Table 1:** The proposed method for ground truth error and its standard deviation error. (a), (b), (c) correspond to the parameters in Figure 10 and Figure 11.

Data Sets	(a)		(b)		(c)	
	E (m)	σ (m)	E (m)	σ (m)	E (m)	σ (m)
ZAE	0.6611	0.7487	0.7142	0.6625	0.4698	0.5282
Parking	0.7116	0.8166	0.6874	0.8039	0.5813	0.6876

**Table 2:** Time calibration error.

Data Sets	Mean [s]	std [s]	RMSE [s]
Computer Science	−0.0374	0.0278	0.0466
ZAEG	−0.0126	0.2518	0.2516
ParkingG	−0.0236	0.1977	0.1984
Campus	−0.0216	0.1967	0.1975
